# A d-dimer and ADAMTS8 based multi-marker score for the diagnosis of acute aortic dissection

**DOI:** 10.1038/s41598-026-51121-w

**Published:** 2026-05-07

**Authors:** Ting Tian, Liping Zhao, Xinxin Tian, Fen Liu, Qiang Zhao, Junyi Luo, Qian Zhao, Yanhong Li, Xiaomei Li, Yining Yang

**Affiliations:** 1 Department of Cardiology, The First Affiliated Hospital of Xinjiang Medical University, 137 Liyushan South Road, Urumqi, 830054 Xinjiang China; 2 State Key Laboratory of Pathogenesis, Prevention and Treatment of High Incidence Diseases in Central Asian, Clinical Medical Research Institute, Xinjiang Medical University, Urumqi, 830054 China; 3 Key Laboratory of Cardiovascular Disease Research, First Affiliated Hospital of Xinjiang Medical University, Urumqi, 830054 China; 4 Department of Cardiology, People’s Hospital of Xinjiang Uygur Autonomous Region, 91 Tianshan Road, Urumqi, 830001 Xinjiang China; 5 Xinjiang Key Laboratory of Cardiovascular Homeostasis and Regeneration Research, Urumqi, 830001 China

**Keywords:** Acute aortic dissection, Diagnostic biomarker, Multi marker score, Gene expression, Risk factors, Biomarkers, Aortic diseases

## Abstract

Acute aortic dissection (AAD) is a life-threatening emergency without established effective monitoring biomarkers. This study aimed to explore biomarkers to optimize the diagnosis of AAD. AAD related genes were screened by spatial transcriptomics experiments, and their encoded proteins were validated in aortic tissues. We measured plasma levels of candidate proteins in 302 participants (173 AAD cases, 129 controls), finding higher PTMA, ADAMTS8, and CD36 in AAD. Case-control analysis revealed that elevated levels of those proteins along with d-dimer, increased systolic blood pressure (SBP), height and smoking history were risk factors for AAD. A multi-marker score comprising d-dimer, ADAMTS8, height, SBP, and age was developed for AAD diagnosis, achieving an AUC of 0.921 (95%CI 0.889–0.952), with 77.5% sensitivity and 96.5% specificity. We further validated the diagnostic performance of the multi-marker score in an independent validation set including healthy controls and patients with chest pain. Our findings indicate that PTMA, ADAMTS8, and CD36 are potential biomarkers associated with AAD. The multi-marker score effectively discriminates AAD from both healthy controls and non-AAD acute chest pain conditions, and may serve as a rapid, cost-effective auxiliary diagnostic tool.

**Supplementary Information:**

The online version contains supplementary material available at10.1038/s41598-026-51121-w.

## Introduction

As a life-threatening cardiovascular disease, acute aortic dissection (AAD) is a rapidly fatal clinical emergency, with high rates of mortality. According to the International Register of Aortic Dissection Studies data^1^, the annual incidence of AAD in European or American countries is about 3–5 cases per 100,000 individuals. In accordance with the prediction of the UK’s Office for National Statistics population projections, the number of the incident dissection events will increase from 3892 in 2010 to 6893 in 2050^2^. In addition, the overall global death rates from AAD have increased during the past two decades and it seems to be more evident in developing countries. Despite important advances in diagnostic and therapeutic interventions, the burden of AAD treatment remains clinically high challenging^3^. The untreated mortality is approximately 1%–2% per hour following symptom onset, and it remains high even after surgical intervention^4,5^. Therefore, early identification and confirmation of a suspected AAD diagnosis, along with timely and appropriate intervention, are crucial in reducing mortality rates^6^. Currently, the diagnosis of AAD mainly relies on imaging techniques including ultrasound, computed tomography (CT), or magnetic resonance imaging (MRI)^7^. However, these methods, while recommended for screening in high-risk populations and for assessing patients at the onset of symptoms, are not suitable for early screening in the general population^3,8,9^. Consequently, there is a pressing need to identify specific biomarkers for AAD in both clinical practice and research, due to the concealment of the disease.

Biomarkers are helpful to diagnose and determine the risk of AAD in a timely and easily available manner^3,10^. Currently, potential biomarkers have been focusing on those associated with pathophysiologic changes in aortic wall, such as smooth muscle markers, which reflect the disintegration and release of medial smooth muscle cellular components into the circulation during dissection^11,12^. Similarly, extracellular matrix proteins (ECM), inflammatory response and markers are also released into the circulation^10^. More biomarkers including cardiac stress or damage, thrombosis or fibrinolysis and inherited predisposition are being researched. d-dimer reflects the dynamic coagulation state and is recognized as an exclusion biomarker for pulmonary embolism. It has a clinically relevant role in suspected AAD and is highly sensitive to AAS. Several studies have shown that d-dimer has a clinically relevant role in suspected AAD^13,14^. However, the specificity of d-dimer ranges from 40 to 100%^6,15^ which may be lower in patients with false lumen thrombosis, less extensive disease and younger age individuals^16^. Moreover, the negative d-dimer test result is not sufficient to exclude AAD, and other meaningful indicators need to be combined to optimize the diagnosis.

The emerging paradigm of precision medicine highlights the importance of quantitative portray of molecular features during disease onset and progression. Dynamic markers, especially the circulating proteins, may help to capture the disease trajectory and to offer precise prognostication for AAD patients^17^. In our previous studies, we have collected AAD tissues and conducted an initial exploration of AAD-related genes by spatial transcriptomics (ST)^18^. To study how genes affect health and disease, and to find novel biomarkers, it is essential to further investigate the expression of proteins encoded by these genes in dissection tissues and blood circulation.

Leveraging the AD resources using ST, we sought to (1) screen the AAD related genes, (2) validate the protein expression encoded by these genes in tissues, (3) confirm protein expression in blood circulation in larger clinical blood samples, (4) develop diagnostic panels to differentiate AAD from healthy controls, and (5) explore the discriminative efficacy in patients with chest pain. We also included an independent validation set to verify the diagnostic performance of the developed panels. We hope that these identified candidate proteins can assist in the clinical diagnosis of AAD and ultimately improve the treatment strategies for this condition.

## Materials and methods

### Study design and patients’ enrollment

**Fig. 1:**
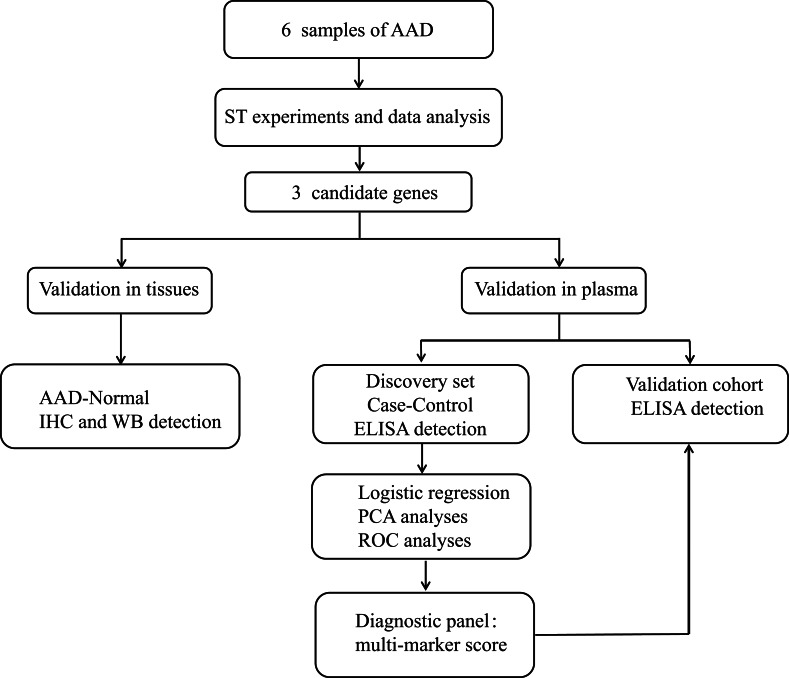
The overall experimental design.

The overall experimental design is summarized in Fig. 1. The human aortic samples used for ST analysis and protein validation were collected from patients undergoing vascular replacement surgery. Control tissues were from organ donation patients. The clinical research is comprised a retrospective discovery set and a validation set. In the discovery set, 173 patients diagnosed with AAD through computed tomography angiography from December 2021 to December 2023 and 129 healthy control patients were included to evaluate the diagnostic performance of candidate molecules in distinguishing AAD from the normal population. In order to further validate the diagnostic value of the screened biomarkers, we designed an independent validation set, including suspected acute AAD patients with chest pain who visited the emergency department from March 2025 to March 2026. Finally, confirmed pulmonary embolism patients (PE) (42 patients), confirmed AAD patients (130 patients), patients with acute myocardial infarction (AMI) (99 patients), along with 122 healthy control patients. Human subjects with the Marfan syndrome, Ehlers-Danlos syndrome, bicuspid aortic valve, malignant tumor, pregnancy and trauma were excluded. Baseline characteristics including medical history, physical examination biochemical data and surgical information were obtained through medical records. All clinical specimens were recruited from the First Affiliated Hospital of Xinjiang Medical University and the People’s Hospital of Xinjiang Uygur Autonomous Region.

Written informed consent forms were obtained from the patients or the donors’ families. The research protocol conforms to the ethical guidelines of the Declaration of Helsinki and was approved by the Ethics Committee of the First Affiliated Hospital of Xinjiang Medical University (20150006-8) and the Ethics Committee of People’s Hospital of Xinjiang Uygur Autonomous Region (KY2022072259).

### ST experiments and data analysis

ST detection of AAD is our previous study^18^. The detailed collection and preparation of aortic samples and the following 10x Genomics and ST sequencing experiments were described in our previous studies^18^. In short, spatial gene expression library was constructed on Illumina’s NOVA 6000 platform. The Seurat package (version 3.1.3) implemented in R software^19^ was used to analyze the gene-spot matrices derived from ST data processing and Visium samples. Perform subsequent analysis using principal component analysis (PCA) and ICA methods, and cluster each point based on K-nearest neighbor algorithm. A spatial cluster gene list was generated for all genes differentially expressed in ST clusters (average logFC > 0.25, *P* value < 0.05, and only retained positively regulated genes). Compare the expression of each gene from one cluster to the spots of all other clusters with the average expression. The DEGs with the largest changes in expression in each cluster were ranked. Cell Marker database^20^, clusterProfiler (version 3.12.0)^21^, and Human Cell Landscape (HCL) database were helped to classify the cell subpopulations and identify the Cell types of different locations.

### Histological analysis

The aorta samples were quickly washed with cold saline solution immediately after surgical resection, then frozen in liquid nitrogen for protein extraction or fixed in 4% paraformaldehyde for 24 h. Following the standard paraffin embedded, cross sections were prepared at 5 μm for immunohistochemical (IHC) staining. The antibodies were as follows: cluster of differentiation 36 molecule (CD36, 1:100, Novus Biologicals, NB400-144), a disintegrin and metalloproteinase with thrombospondin motifs 8 (ADAMTS8, 1:100, Abnova, PAB18816) and prothymosin α (PTMA, 1:200, ABGENT, AP11770a). The images were captured using a Leica microscope system. For quantitative analysis, Image-Pro Plus 6.0 software was used to evaluate the area and the integrated optical density (IOD) value of each IHC sections and calculate the mean density.

### Protein extraction and western blot analysis

Extract total protein from human aortas using the Minute™ Total Protein Extraction Kit for Blood Vessels (SA-03-BV, Invent Biotechnologies). Standard sodium dodecyl sulfate polyacrylamide gel electrophoresis (SDS-PAGE) protocols and PVDF membranes were employed to detect target protein expression. The following primary antibodies were used: CD36 (1:1000, Abcam, ab252923), ADAMTS8 (1:300, Abnova, PAB18816) and PTMA (1:1000, ABGENT, AP11770a). Glyceraldehyde-3-phosphate dehydrogenase (GAPDH, 1:1000, Cell Signaling, 5174s) was used as loading control.

### Collection of plasma samples and measurements of target proteins

Blood samples were obtained from participants and collected from AAD patients before surgery. After centrifuged, plasma was immediately processed and stored at − 80 °C before measured. Circulating target proteins were measured using ELISA double antibody sandwiched method (Jianglai Bio., Shanghai, China) according to the manufacturer’s instructions. To ensure accuracy, all samples were analyzed in duplicate, and both intra- and inter-assay coefficients of variation were maintained at less than 10%.

### Clinical data collection

We collected the clinical information from patients’ electronic medical records, including age, gender, symptom, body mass index, blood pressure, heart rate, history of smoking, hyperlipidemia and hypertension. Biochemical and hematological data were also recorded.

### Statistical analysis

Continuous variables were expressed as mean with standard deviation (SD) or median with interquartile range (IQR) for skewed variables. We used the unpaired Student’s t-test or the Mann-Whitney U test to compare variables between groups. Categorical variables are presented as frequencies and percentages and were conducted with chi-square test. Univariate and multivariate Logistic regression analyses were performed to identify independent risk factors for AAD. Results were presented as odds ratio (OR) with 95% confidence interval (CI).

PCA was utilized to figure out the distribution pattern of epidemiological risk factors between healthy subjects or AAD patients, while identifying the most important variables contributing to different patterns (ranked by PCA loadings). For all proteins and clinical parameters, we selected biomarkers to develop the AAD diagnostic multi-marker score system through PCA loadings, according to the optimal predictive thresholds from receiver operating characteristic (ROC) analysis and practice guidelines: d-dimer ≥ 500 ng/mL^22^, ADAMTS8 ≥ 802.5 pg/mL, Height ≥ 166.5 cm, SBP ≥ 140 mmHg^2,23^ and Age ≥ 65 years^3,6^. 1 point was assigned to each selected biomarker described above and multiple biomarker scores was generated from 0 to 5. We used regression curves with restricted cubic spline analysis to check the relationship between multi-marker score as a continuous variable and AAD events. ROC curve and area under the curve (AUC) were calculated and compared to assess the diagnostic performance and predictive capability of the multi-marker score.

Statistical analyses were performed in SPSS (version 26.0) and R statistical software version 4.0.3 (R Foundation). Differences were considered as significant for *P* values < 0.05.

## Results

### Screening for novel candidate genes related to AAD

**Fig. 2:**
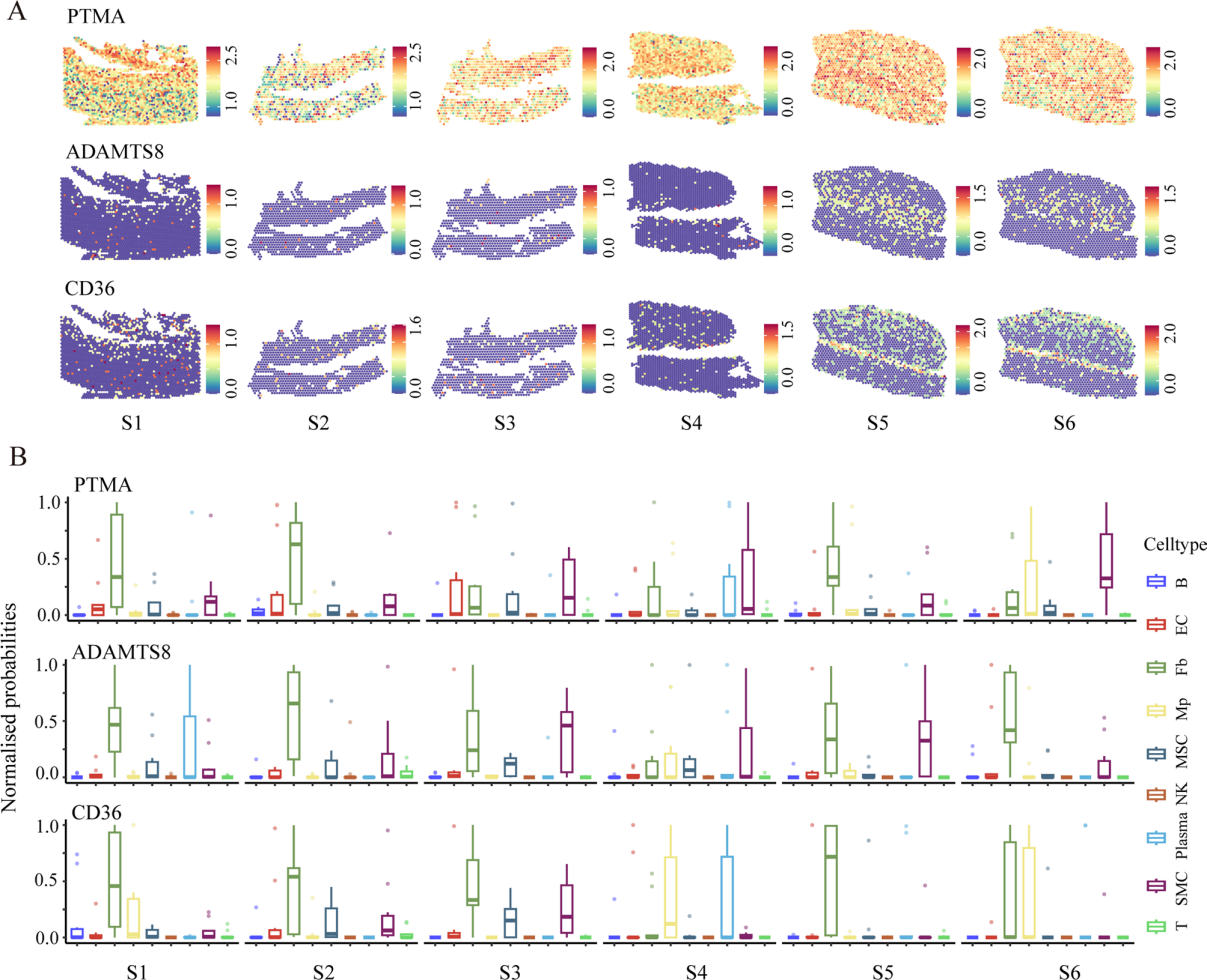
Selection of candidate proteins related to AAD. (**A**) Spatial distribution maps of three candidate genes PTMA, CD36 and ADAMTS8 selected by spatial transcriptomics (ST) experiments in each section (n = 6). (**B**) Cellular composition in ten spots exhibiting the highest gene expression. EC, endothelial cell; Fb, fibroblasts; Mp, macrophages; MSC, mesothelial cell or mesenchymal-stromal cell; NK, natural killer cell; SMC, smooth muscle cell; T, T cells.

In our previous studies involving ST experiments of AAD, a spatial cluster gene list was generated for differentially expressed in ST clusters (average logFC > 0.25, *P* value < 0.05, and only retained positively regulated genes) (Supplemental: Table S1). To identify novel genes related to AAD, we employed the following selection criteria: (1) expression in the artery and the heart, (2) encoding of a secretory protein, (3) association with pathological mechanisms of AAD such as inflammation, cell apoptosis, or vascular homeostasis regulation, and (4) few reported by literature search. Utilizing these criteria, we ultimately selected three candidate genes *ADAMTS 8* (encoding ADAMTS8), *CD36* (encoding CD36), and *PTMA* (encoding PTMA) (Fig. 2A).

Considering the varied expression of *ADAMTS8*,* CD36*, and *PTMA* in different cell types, in order to clarify the cell types with high expression regions of *ADAMTS8*,* CD36*, and *PTMA*, we further analyzed ten spots exhibiting the highest gene expression to assess cellular composition. Combined with the cell marker database^24^, clusterProfiler tool, and AAD related cell markers, such as VSMC marker α-smooth muscle actin (α-SMA), endothelial cell marker cluster of differentiation 31 (CD31), macrophage marker cluster of differentiation 68 (CD68)^25^, etc. the cell markers at the sites were identified. This analysis identified several cell types associated with each gene, and the results showed that the cell composition of *ADAMTS8*,* CD36*, and *PTMA* high expression sites mainly included fibroblasts, vascular smooth muscle cells (VSMCs), mesenchymal stromal cells, macrophages, and endothelial cells (Fig. 2B). The above results indicate that *ADAMTS8*,* CD36*, and *PTMA* may participate in the occurrence and development of AAD by regulating the functions of multiple cell types, including VSMCs, fibroblasts, and macrophages.

### Validation of candidate proteins in tissues

**Fig. 3:**
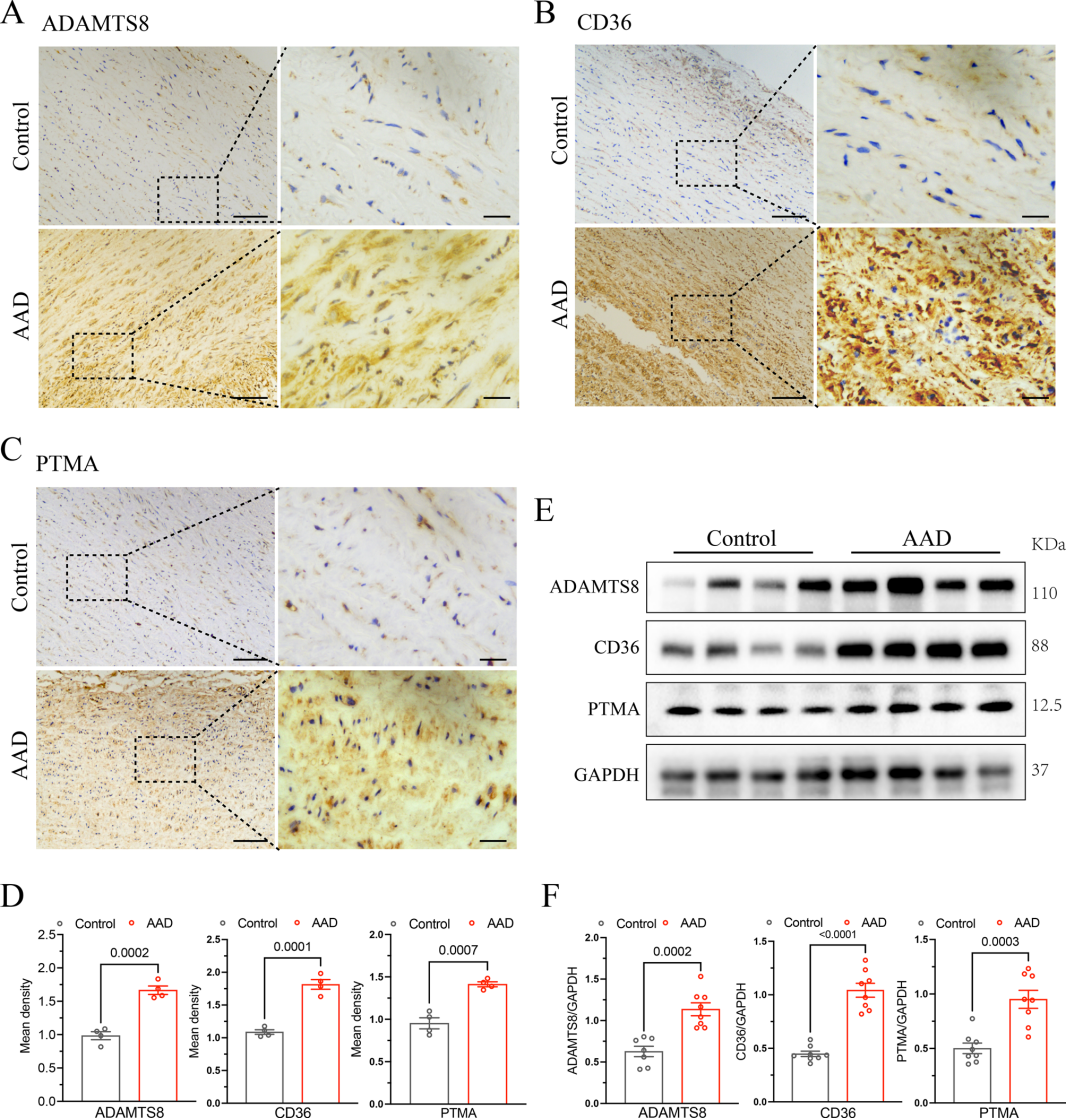
Validation of candidate proteins related to AAD in aortic tissues. Candidate protein expression was validated by immunohistochemistry (IHC) and Western blot (WB) detection. (**A-D**) Representative IHC results of PTMA, CD36, and ADAMTS8 in aortic tissues from AAD patients and normal controls respectively. (n = 4/group). Scale bars are 50 µm and 20 µm for the zoom image. (**E**, **F**) Representative Western blot images and quantification of PTMA, CD36, ADAMTS8, and GAPDH in AAD tissues and normal aortic tissues (n = 4/group).

To confirm the expression levels of the coding proteins ADAMTS8, CD36 and PTMA corresponding to the candidate genes screened above, we utilized aorta tissues from AAD patients for both IHC and Western blot detection. The IHC results of the aorta showed that compared with the healthy control group, the positive staining area and average optical density of PTMA, ADAMTS8, and CD36 in the AAD aorta were significantly increased ( all *P* < 0.05) (Fig. 3A–D). The Western blot results of the aorta showed that compared with the healthy control group, the relative expression levels of these three candidate proteins in AAD aorta were significantly upregulated ( all *P* < 0.05) (Fig. 3E, F). These results suggest that PTMA, ADAMTS8 and CD36 are abnormally overexpressed in the aortic wall of AAD lesions, and their expression changes may be closely related to the occurrence and development of AAD.

### Clinical synopsis and proteins distribution in plasma

**Fig. 4:**
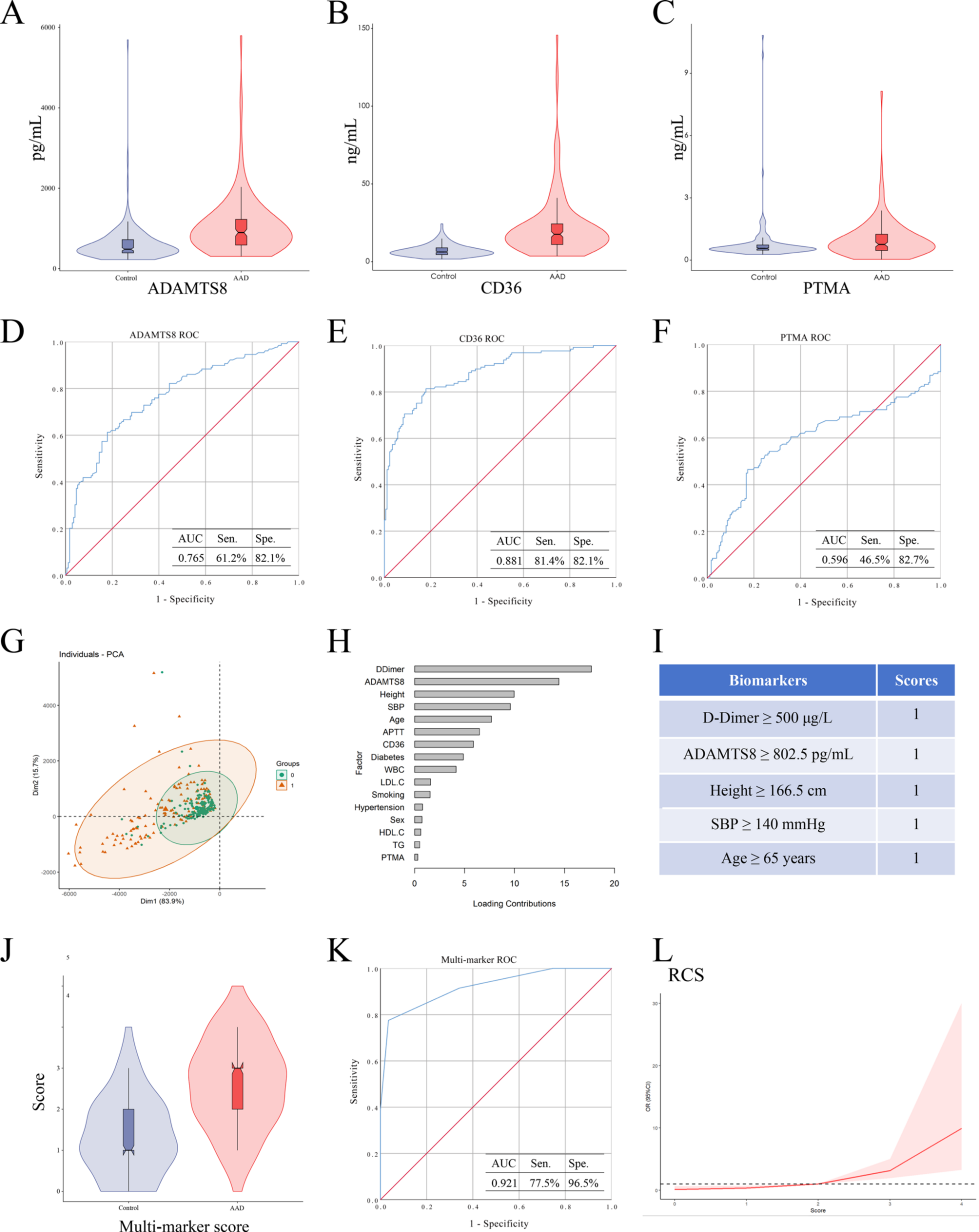
Exploration of protein distribution in plasma and development of a multi-marker score. (**A**-**C**) The expression levels of PTMA, ADAMTS8, and CD36 proteins were significantly higher in AAD patients than in control subjects. ADAMTS8 levels were elevated to 898.00 pg/mL (median, IQR: 575.50 – 1241.00 vs. 484.00 pg/mL (median, IQR: 392.00 – 726.50) in healthy controls (P < 0.0001), CD36 levels were elevated to 17.59 ng/mL (median, IQR: 10.72 – 24.39) vs. 6.25 ng/mL (median, IQR: 4.54 – 8.91) in healthy controls (P < 0.0001), and PTMA levels were elevated to 0.76 ng/mL (median, IQR: 0.46 – 1.28) vs. 0.57 ng/mL (median, IQR: 0.48 – 0.74) in healthy controls (P = 0.004). (**D**-**F**) ROC curve analysis of individual proteins: ADAMTS8 (AUC=0.782, 95%CI: 0.733-0.831), CD36 (AUC=0.881, 95%CI: 0.842-0.920), and PTMA (AUC=0.596, 95%CI: 0.527-0.665). (G) PCA plot based on the top two principal components distinguishing AAD group from healthy controls. (**H**) Selection of five core predictive factors (D-dimer, ADAMTS8, height, SBP and age) based on PCA loading values. (**I**) Optimal cut-off values of the five core factors for multi-marker score construction: D-dimer ≥ 500 ng/mL, ADAMTS8 ≥ 802.5 pg/mL, height ≥ 166.5 cm, SBP ≥ 140 mmHg, age ≥ 65 years. (**J**) Multi-marker scores were significantly higher in the AAD patients than in the controls. (**K**) ROC curve analysis of the multi-marker score for AAD diagnosis (AUC = 0.921, 95%CI: 0.889 - 0.952; sensitivity 77.5%, specificity 96.5%). (**L**) Restricted cubic spline regression showing the relationship between multi-marker score and AAD events, with exponentially increased AAD risk when the score ≥ 3 points.

A total of 302 participants participated were enrolled in this discovery set, comprising 173 AAD cases and 129 controls (Table 1). The mean age of AAD patients was 53.79 ± 10.35 years, which was comparable to the mean age of 53.47 ± 12.45 years in controls (*P* = 0.808). Men were accounted for 79.8% in AAD patients and for 69.9% in controls (*P* = 0.052). The AAD patients exhibited significantly higher levels of the independent risk factors including systolic blood pressure (SBP), smoking, hypertension, standing height and d-dimer levels. Subsequently, the circulating levels of PTMA, ADAMTS8, and CD36 were measured in plasma samples from patients with AAD and healthy control subjects. Plasma concentrations of all three candidate proteins were significantly elevated in the AAD group compared with healthy controls. ADAMTS8 levels were elevated at 898.00 pg/mL (median, IQR: 575.50–1241.00) vs. 484.00 pg/mL (median, IQR: 392.00–726.50) (*P* < 0.0001); CD36 levels were elevated at 17.59 ng/mL (median, IQR: 10.72–24.39) vs. 6.25 ng/mL (median, IQR: 4.54–8.91) (*P* < 0.0001); and PTMA levels were elevated at 0.76 ng/mL (median, IQR: 0.46–1.28) vs. 0.57 ng/mL (median, IQR: 0.48–0.74) (*P* = 0.004). (Fig. 4A–C). These results suggest significant differences in the expression of these three candidate molecules in peripheral blood, which can serve as potential molecular markers for AAD diagnosis and prediction.

**Table 1:** Patient characteristics in the control group and AAD group.

Characteristics	Control group (*n* = 173)	AAD group (*n* = 129)	t/Z/χ²	*P* value
Characteristics demographics				
Age (y)	53.79 ± 10.35	53.47 ± 12.45	0.243	0.808
Male sex, n (%)	121 (69.90)	103 (79.80)	3.783	0.052
Height (cm)	165.00 (160.00, 174.00)	173.00 (168.00, 175.00)	− 5.18	< 0.0001
BMI (kg/m^2^)	25.88 (24.10, 28.08)	26.17 (22.95, 28.55)	− 0.22	0.823
SBP (mmHg)	124.00 (115.00, 138.00)	136.00 (120.00, 160.00)	− 5.01	< 0.0001
DBP (mmHg)	78.00 (71.00, 84.00)	80.00 (69.00, 87.50)	− 0.51	0.612
HR (BPM)	76.00 (70.00, 82.00)	81.00 (73.00, 93.00)	− 3.25	0.001
Medical history and risk factors				
Hypertension, n (%)	87 (50.30)	104 (80.60)	29.25	< 0.0001
Diabetes, n (%)	18 (10.40)	19 (14.70)	1.29	0.169
Smoking, n (%)	43 (24.90)	62 (48.10)	17.55	< 0.0001
Biochemical and hematological data				
White blood cell, 10*9/L	6.36 (5.22, 7.41)	8.55 (6.59, 10.89)	− 2.495	0.018
FPG (mmol/L)	4.92 (4.48, 5.90)	6.66 (5.63, 7.83)	− 7.65	< 0.0001
TC (mmol/L)	4.10 ± 1.15	4.14 ± 0.93	− 0.58	0.564
TG (mmol/L)	1.51 (1.05, 2.11)	1.35 (1.02, 1.88)	− 1.02	0.307
HDL-C (mmol/L)	1.02 (0.86, 1.20)	0.84 (0.72, 0.99)	− 5.84	< 0.0001
LDL-C (mmol/L)	2.55 ± 0.88	2.76 (2.42, 3.10)	− 0.96	0.337
d-dimer (µg/L)	72.50 (43.00, 131.75)	1019.00 (467.00, 2896.00)	− 13.03	< 0.0001
ADAMTS8 (pg/mL)	484.00 (392.00, 726.50)	898.00 (575.50, 1241.00)	− 7.87	< 0.0001
CD36 (ng/mL)	6.25 (4.54, 8.91)	17.59 (10.72, 24.39)	− 11.34	< 0.0001
PTMA (ng/mL)	0.57 (0.48, 0.74)	0.76 (0.46, 1.28)	− 2.86	0.004

*BMI* Body mass index, *SBP* Systolic blood pressure, *DBP* diastolic blood pressure, *HR* heart rate, *BPM* beats per minute, *FPG* Fasting plasma glucose, *TC* total cholesterol, *TG* triglyceride, *HDL-C* High density lipoprotein cholesterol, *LDL-C* Low density lipoprotein cholesterol, *CD36* cluster of differentiation 36 molecule, *ADAMTS8* a disintegrin and metalloproteinase with thrombospondin motifs 8, *PTMA* prothymosin α.

### Plasma ADAMTS8, CD36 and d-dimer are independent risk factors for AAD

Univariate logistic regression analysis identified that plasma protein markers such as ADAMTS8 and CD36, clinical characteristics such as sex, height, SBP, and smoking history, and laboratory indicators such as white blood cells, d-dimer, and high-density lipoprotein cholesterol (HDL-C) were significantly correlated with the risk of AAD (all *P* < 0.05). Among these, ADAMTS8 (OR = 6.774, 95% CI 3.421–13.142, *P* < 0.0001), CD36 (OR = 1.316, 95% CI 1.233–1.405, *P* < 0.0001), and d-Dimer (OR = 1.011, 95% CI 1.008–1.014, *P* < 0.0001) were identified as independent risk factors for AAD, whereas HDL-C (OR = 0.042, 95% CI 0.013–0.129, *P* < 0.0001) was a protective factor. Multivariate logistic regression analysis further demonstrated that after adjustment for potential confounders including sex, SBP, heart rate, smoking history, white blood cell, serum creatinine, and triglycerides, ADAMTS8 (aOR = 4.596, 95% CI 1.250–19.890, *P* = 0.022), CD36 (aOR = 1.521, 95% CI 1.244–1.860, *P* < 0.0001), and d-dimer (aOR = 1.013, 95% CI 1.007–1.019, *P* < 0.0001) remained independent risk factors for AAD. In contrast, HDL-C (aOR = 0.001, 95% CI 0.001–0.028, *P* = 0.002) remained an independent protective factor. These details were summarized in Table 2.

**Table 2:** Univariate and multivariate analysis and epidemiological risk factors for AAD.

Variables	Univariate analysis		Multivariate analysis	
	Odds ratio (95% CI)	*P* value	Odds ratio (95% CI)	*P* value
ADAMTS8	6.774 (3.421, 13.412)	< 0.0001	4.596 (1.250, 16.896)	0.022
CD36	1.316 (1.233, 1.405)	< 0.0001	1.521 (1.244, 1.860)	< 0.0001
PTMA	1.206 (0.944, 1.541)	0.134		
Age	0.997 (0.977, 1.018)	0.808		
Male sex	3.570 (2.115, 6.026)	< 0.0001	1.476 (0.065, 33.537)	0.807
Height	1.086 (1.050, 1.124)	< 0.0001	1.046 (0.962, 1.137)	0.186
SBP	1.030 (1.018, 1.043)	< 0.0001	1.001 (0.975, 1.028)	0.296
DBP	1.002 (0.984, 1.021)	0.803		
HR	1.028 (1.008, 1.047)	0.005	1.035 (0.991, 1.081)	0.117
Diabetes	1.487 (0.746, 2.964)	0.259		
Smoking	2.798 (1.717, 4.558)	< 0.0001	1.793 (0.279, 11.513)	0.538
White blood cell	1.332 (1.201, 1.477)	< 0.0001	1.297 (0.975, 1.724)	0.074
Creatinine	1.037 (1.023, 1.051)	< 0.0001	1.020 (0.992, 1.049)	0.153
TG	0.842 (0.666, 1.064)	0.150		
HDL-C	0.042 (0.013, 0.129)	< 0.0001	0.001 (0.001, 0.028)	0.002
d-dimer	1.011 (1.008, 1.014)	< 0.0001	1.013 (1.007, 1.019)	< 0.0001

*BMI* Body mass index, *SBP* Systolic blood pressure, *DBP* diastolic blood pressure, *HR* heart rate, *FPG* Fasting plasma glucose, *TG* triglyceride, *HDL-C* High density lipoprotein cholesterol, *LDL-C* Low density lipoprotein cholesterol, *CD36* cluster of differentiation 36 molecule, *ADAMTS8* a disintegrin and metalloproteinase with thrombospondin motifs 8, *PTMA* prothymosin α.

### Diagnostic value of plasma proteins in AAD

Based on the genomic and protein validations related to AAD in tissues and plasma, we aimed to search for identify specific protein combinations as potential diagnostic panels for AAD. To assess the accuracy of the three candidate proteins as diagnostic biomarkers, the ROC analysis was conducted to evaluate their sensitivity and specificity individually. The AUC values were 0.765 (95%CI 0.710–0.819), 0.881 (95%CI 0.842–0.920) and 0.596 (95%CI 0.527–0.666) for ADAMTS8, CD36 and PTMA, respectively (Fig. 4D–F). Candidate proteins with an AUC > 0.7 may be considered as diagnostic biomarkers for AAD.

### AAD risk pattern recognition and variable screening

To achieve precise identification of AAD risk patterns and screen for core diagnostic variables, this study integrated molecular markers with significant differential expression at both tissue and plasma levels, and combined key clinical indicators to construct an AAD specific diagnostic model. PCA was performed using independent predictors of AAD validated by logistic regression, including ADAMTS8, d-dimer, and related clinical variables, with varimax orthogonal rotation for component extraction. The results showed that the variance contribution rate of principal component 1 was 83.9%, and that of principal component 2 was 15.7%. The cumulative variance contribution rate of the top two principal components reached 99.6%, indicating that these two components captured 99.6% of the effective information from the original variables and adequately reflect the core risk characteristics of AAD pathogenesis (Supplemental: Fig. 1).

PCA plots based on principal components 1 and 2 can clearly distinguish the AAD group from healthy controls (Fig. 4G). These findings further validate that the PCA model incorporating ADAMTS8, d-dimer, and clinical variables can effectively identify AAD risk patterns, confirming the discriminatory value of the selected key variables for AAD.

### Develop multi-marker score for AAD

Based on the two principal component analysis results with a cumulative variance contribution rate of 99.6%, the five core predictive factors with the highest contribution to the AAD risk model were selected according to the PCA load value, including two biomarkers: d-dimer, ADAMTS8, and three clinical variables: height, systolic blood pressure, and age (Fig. 4H). The optimal threshold determined based on clinical diagnosis and treatment guidelines and experimental data is set as follows: d-dimer ≥ 500 ng/mL, ADAMTS8 ≥ 802.5 pg/mL, height ≥ 166.5 cm, SBP ≥ 140 mmHg (according to the diagnostic criteria for hypertension), and age ≥ 65 years old. The total score range of this scoring system is 0–5 points, with higher scores indicating a higher risk of AAD in the research subjects (Fig. 4I).

The multi-marker score was significantly higher in AAD patients than in controls (Fig. 4J). The sensitivity and specificity of multi-marker score for AAD diagnosis were 77.5% and 96.5% with an AUC of 0.921 (95%CI 0.889–0.952) (Fig. 4K). Regression analysis with restricted cubic spline described the relationship between multi-marker score as a continuous variable and AAD events, suggesting that the likelihood of AAD events increased with the multi-marker score, and the risk increased exponentially when the score reached ≥ 3 points (Fig. 4L).

### Validation of multi-marker score in validation set

To validate the diagnostic and predictive value of the multi-marker score for AAD, we established an independent validation set comprising 393 participants: 271 patients with acute chest pain (130 AAD cases, 99 AMI cases, and 42 PE cases) and 122 healthy controls. Baseline characteristics are presented in Table 3.

**Table 3:** Patient characteristics in the validation set.

Characteristics	Healthy group (*n* = 122)	AAD group (*n* = 130)	AMI group (*n* = 99)	PE group (*n* = 42)
Characteristics demographics				
Age (y)	52.02 ± 10.54	52.45 ± 12.65	58.92 ± 12.65	52.45 ± 9.20
Male sex, n (%)	84 (68.85)	102 (78.46)	87 (87.88)	20 (47.62)
Height (cm)	170.00 (165.00, 175.00)	171.00 (165.00, 176.00)	172.00 (165.00, 176.00)	164.00 (159.75, 172.00)
BMI (kg/m2)	26.50 (24.59, 29.99)	26.82 (24.60, 29.58)	27.17 (24.34, 30.19)	25.16 (23.63, 29.44)
SBP (mmHg)	133.50 (122.00, 143.00)	145.00 (129.75, 162.00)	122.00 (110.00, 137.00)	123.50 (115.00, 136.25)
DBP (mmHg)	80.00 (73.00, 89.25)	75.00 (62.00, 89.25)	75.00 (65.00, 82.00)	75.00 (68.75, 83.25)
HR (BPM)	79.00 (74.00, 88.00)	79.00 (71.00, 88.00)	86.00 (75.00, 94.00)	85.50 (78.00, 93.50)
Medical history and risk factors				
Hypertension, n (%)	100 (18.03)	101 (22.31)	38 (61.62)	19 (54.76)
Diabetes, n (%)	94 (77.05)	122 (93.85)	73 (72.73)	34 (80.95)
Smoking, n (%)	85 (69.67)	99 (76.15)	56 (56.57)	33 (78.57)
Biochemical and hematological data				
White blood cell, 10*9/L	6.57 (5.75, 7.77)	11.82 (9.35, 14.51)	11.28 (8.28, 14.35)	7.39 (6.64, 9.64)
TC (mmol/L)	3.99 (3.32, 4.75)	3.59 (3.11, 4.24)	4.53 (3.58, 5.43)	2.59 (1.57, 3.24)
TG (mmol/L)	1.51 (1.00, 2.12)	1.16 (0.82, 1.53)	1.47 (1.07, 2.50)	1.49 (0.85, 3.18)
HDL-C (mmol/L)	0.93 (0.81, 1.10)	1.02 (0.82, 1.28)	1.07 (0.89, 1.24)	0.98 (0.83, 1.27)
LDL-C (mmol/L)	2.40 (1.91, 3.07)	2.25 (1.91, 2.55)	2.92 (2.25, 3.66)	2.07 (1.43, 2.91)
d-dimer (µg/L)	97.50 (72.50, 150.00)	1452.50 (819.75, 2884.0)	245.00 (120.64, 391.26)	1218.00 (632.25, 2394.00)
ADAMTS8 (pg/ml)	504.03 (394.40, 838.89)	913.33 (733.86, 1067.66)	622.50 (431.95, 732.08)	660.48 (524.13, 760.44)
Multi-scores, n (%)				
0	6 (4.92)	0 (0.00)	6 (7.07)	0 (0.00)
1	62 (50.82)	3 (2.31)	51 (51.52)	7 (16.67)
2	48 (39.34)	24 (18.46)	32 (32.32)	26 (61.90)
3	6 (4.92)	58 (44.62)	10 (10.10)	8 (19.05)
4	0 (0.00)	43 (33.08)	0 (0.00)	1 (2.38)
5	0 (0.00)	2 (1.54)	0 (0.00)	0 (0.00)

**Fig. 5:**
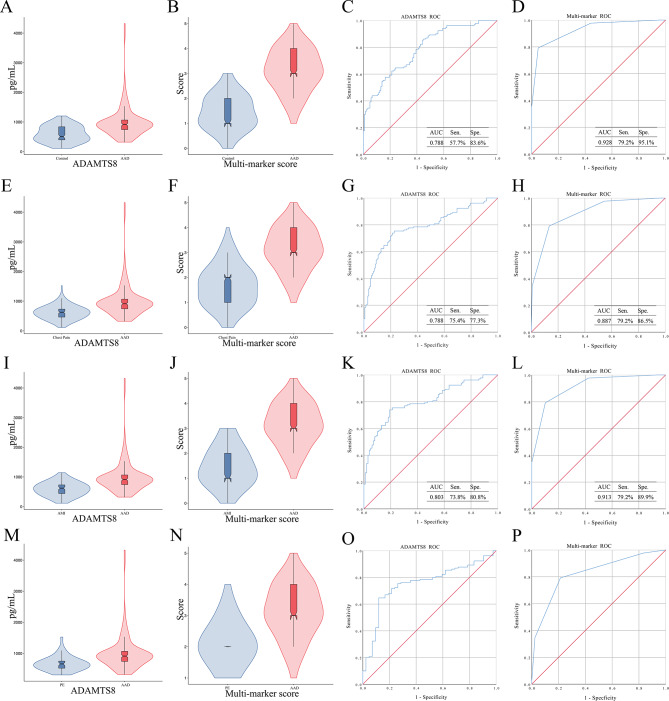
Validation of the ADAMTS8 levels and the multi-marker score in the independent validation set. (**A**, **B**) ADAMTS8 levels and multi-marker scores were significantly elevated in AAD group compared with healthy controls (ADAMTS8, median [IQR]: 913.33 [733.86, 1067.66] pg/mL vs. 504.03 [394.40, 838.89] pg/mL, P < 0.0001). (**C**) ROC curve of ADAMTS8 for differentiating AAD from healthy controls (AUC = 0.788, 95% CI: 0.733–0.842). (**D**) ROC curve analysis of the multi-marker score for discriminating AAD from healthy controls (AUC = 0.928, 95% CI: 0.897–0.959). (**E**, **F**) ADAMTS8 levels and multi-marker scores were significantly higher in AAD patients than in non-AAD acute chest pain patients (ADAMTS8, median [IQR]: 913.33 [733.86, 1067.66] pg/mL vs. 626.30 [462.80, 732.34] pg/mL, P < 0.0001). (**G**) ROC curve of ADAMTS8 for discriminating AAD from non-AAD acute chest pain patients (AUC = 0.788, 95% CI: 0.732–0.843). (**H**) ROC curve of ADAMTS8 for discriminating AAD from non-AAD acute chest pain patients (AUC = 0.887, 95% CI: 0.848–0.926). (**I**, **J**) ADAMTS8 levels and multi-marker scores were significantly higher in the AAD group than in AMI patients. (ADAMTS8, median [IQR]: 913.33 [733.86, 1067.66] pg/mL vs. 504.03 [394.40, 838.89] pg/mL, P < 0.0001). (**K**, **L**) For differentiating AAD from AMI, ADAMTS8 had an AUC of 0.803 (95% CI: 0.747–0.859), while the multi-marker score reached an AUC of 0.913 (95% CI: 0.877–0.949). (**M**, **N**) ADAMTS8 levels and multi-marker scores were significantly higher in the AAD group than in PE patients. (ADAMTS8, median [IQR]: 913.33 [733.86, 1067.66] pg/mL vs. 660.48 [524.13, 760.44] pg/mL, P < 0.0001). (**O**, **P**) For differentiating AAD from PE, ADAMTS8 had an AUC of 0.752 (95% CI: 0.671–0.833), while the multi-marker score reached an AUC of 0.825 (95% CI: 0.756–0.894).

In the first case-control comparison, both ADAMTS8 levels and multi-marker scores were significantly higher in the AAD group than in healthy controls (Fig. 5A, B). For ADAMTS8, the AUC for differentiating AAD from healthy controls was 0.788 (95% CI 0.733–0.842), with a sensitivity of 57.7% and a specificity of 83.6% (Fig. 5C). For the multi-marker score, the AUC was 0.928 (95% CI 0.897–0.959), with a sensitivity of 79.2% and a specificity of 95.1% (Fig. 5D).

In the second case-control design aimed at distinguishing AAD from non-AAD acute chest pain conditions (AMI and PE), ADAMTS8 levels in AAD patients were 913.33 pg/mL (median, IQR: 733.86–1067.66), which were significantly higher than the 626.30 pg/mL (median, IQR: 462.80–732.34) observed in non-AAD chest pain patients (*P* < 0.0001) (Fig. 5E). The multi-marker score was also markedly elevated in AAD patients(Fig. 5F). For discriminating AAD from all non-AAD acute chest pain patients, ADAMTS8 yielded an AUC of 0.788 (95% CI 0.732–0.843), with 75.4% sensitivity and 77.3% specificity, whereas the multi-marker score achieved an AUC of 0.887 (95% CI 0.848–0.926), with 79.2% sensitivity and 86.5% specificity (Fig. 5G, H). Further stratified analyses showed that for differentiating AAD from AMI, ADAMTS8 had an AUC of 0.803 (95% CI 0.747–0.859; 73.8% sensitivity, 80.8% specificity), while the multi-marker score reached an AUC of 0.913 (95% CI 0.877–0.949; 79.2% sensitivity, 89.9% specificity) (Fig. 5I–L). For distinguishing AAD from PE, ADAMTS8 exhibited an AUC of 0.752 (95% CI 0.671–0.833; 64.6% sensitivity, 88.1% specificity), and the multi-marker score had an AUC of 0.825 (95% CI 0.756–0.894; 79.2% sensitivity, 78.6% specificity) (Fig. 5M–P). Thus, the multi-marker score exhibited superior overall diagnostic performance in differentiating AAD from other conditions presenting with sudden-onset severe chest pain encountered in the emergency department.

## Discussion

In the current study, we identified *PTMA*,* ADAMTS8 and CD36* as genes related to AAD and evaluated cell types in the high expression sites of genes. Compared to the controls, AAD patients showed increased expression of these encoded proteins in the aortic arteries and blood circulation. Through case-control analysis, we demonstrated that elevated SBP, taller standing height and smoking as major epidemiologic risk factors for AAD. We further developed a multi-marker score integrating d-dimer, ADAMTS8, age, height, and SBP. The model was validated in a large independent validation set of 393 subjects, including AAD, AMI, PE, and healthy controls, and showed excellent ability to distinguish AAD not only from healthy individuals but also from non-AAD acute chest pain etiologies. This panel may facilitate early and differential diagnosis of AAD in the emergency setting.

Despite the lethality of AAD, the determinants are still largely unknown. The aortic wall, comprising dynamic cell populations and ECM, responds to biomechanical changes by modulating structure and function, thereby strengthening the aortic wall and meeting hemodynamic demands^26^. Compromised aortic integrity is a fundamental component of the underlying pathology of AAD. While degeneration mechanisms include progressive SMCs depletion, ECM destruction and inflammation, the precise AAD trigger remains elusive. The ST experiments helped us search for novel genes related to AAD. Finally, PTMA, ADAMTS8 and CD36 were selected as candidate proteins for discriminating AAD from controls.

PTMA is a small, widely distributed acidic nuclear protein with cytoplasmic and extracellular localization under specific physiological or pathological conditions^27,28^. Studies have shown that the major intracellular role of PTMA is associated with cell proliferation^28^. Extracellularly, it plays a role in immunomodulation via a pleiotropic mode by stimulating a variety of immune cells^28^. PTMA is also involved in fibrosis by regulating the expression ECM-related markers and the downstream genes of transforming growth factor beta1 (TGF-β1) pathway^29^. Relevant studies have further clarified the regulatory role of PTMA in vascular diseases: it has been identified as a key mediator in vascular remodeling during transplant arteriosclerosis, where it modulates estrogen receptor transcriptional activity and promotes cyclin D1‑dependent vascular cell proliferation^30^. Given that vascular remodeling and abnormal cell proliferation are core pathological features of AAD, the regulatory effect of PTMA on vascular cell proliferation and intimal hyperplasia, as reported in the literature, strongly suggests its potential involvement in aortic wall remodeling and AAD progression, which is consistent with our findings that PTMA is abnormally overexpressed in AAD tissues and plasma.

CD36 is a scavenger receptor densely expressed on the surface of macrophages. Its role in immunity, metabolism and atherosclerosis has been widely studied. CD36 mediates oxidized low-density lipoprotein uptake leading to foam cell formation and inhibits macrophage migration that promotes the trapping of lipid-laden macrophages in the arterial intima^31^. Emerging evidence also indicates that CD36 drives fatty acid-induced ferroptosis in CD4+ T cells in acute type A AD, leading to immune dysfunction and vascular inflammation, excessive reactive oxygen species accumulation, and vascular inflammation^32^. Combined with our findings that CD36 is significantly upregulated in AAD tissues and plasma, and its loss can inhibit VSMC proliferation and regulate contractile protein expression, these literature-based mechanistic insights further confirm that CD36 participates in AAD pathogenesis by linking metabolic disturbance, immune dysregulation, and vascular structural instability. CD36 may contribute to aortic aneurysm development by affecting fatty acid transport and adventitia degradation in the aortic wall. The loss of CD36 has been proven to significantly inhibit VSMC proliferation, increase the expression of TGF-β1 and the contractile proteins including calponin 1 and smooth muscle α actin^33^. These observations collectively suggest that CD36 links metabolic disturbance, immune dysregulation, and structural instability in AAD.

ADAMTS family members, including ADAMTS8, are crucial in vascular homeostasis and vasculopathy, influencing cell proliferation, adhesion, migration and signaling^34–36^. ADAMTS8 is proven to increase SMC proliferation and degrade proteoglycans to promote ECM remodeling^37^. It also induces endothelial dysfunction and matrix metalloproteinase activation in an autocrine/paracrine manner^34^. In line with our present findings in AAD, Levula et al. ^38^ demonstrated that ADAMTS8 expression was significantly higher in ascending AD tissues than in aortic aneurysm (AA) tissues, accompanied by prominent medial inflammation and active vascular remodeling. Similarly, Duma et al.^39^ reported that *ADAMTS8* expression was markedly elevated in the aneurysmal sac of abdominal aortic aneurysm (AAA) compared with non-aneurysmal border segments, supporting a role for *ADAMTS8* in localized pathological matrix degradation^40^. However, a study focused on macrophage transcriptomics and plasma proteomics in AAA patients, found a slight reduction in ADAMTS8 (ratio 0.9) in macrophages and plasma relative to peripheral arterial occlusion controls^41^. This discrepancy may reflect differences in disease subtype (AAD vs. AAA), sample source (tissue vs. macrophage vs. plasma), disease stage, or cellular origin. Moreover, the latest genome-wide association study based on the Million Veteran Program suggests *ADAMTS8* may be a putative causal gene for AAD^42^. Although the exact pathogenic mechanisms of these proteins in AAD are not fully understood, their roles in ECM remodeling and cellular function indicate their potential significance in AAD pathogenesis^34^. Despite variable expression patterns across studies, dysregulated ADAMTS8 whether upregulated or downregulated consistently disrupts ECM homeostasis and compromises aortic wall stability, a core pathological mechanism shared by both aortic dissection and aneurysm. Collectively, our results and existing clinical evidence strongly support that ADAMTS8 is closely implicated in the pathogenesis of AAD. Given the heterogeneous expression profiles and limited mechanistic insights, ADAMTS8 represents a biologically relevant and valuable target for further in-depth investigation in aortic diseases.

When focusing on the dynamics of aortic cells, SMCs are the primary component of the aortic wall and play a central role in maintaining aortic functions and homeostasis^26^. Other cell types including endothelial cells, fibroblasts and myofibroblasts also play vital roles in maintaining the structure and integrity of the aortic wall. Additionally, various inflammatory cells, such as lymphocytes, macrophages, mast cells and neutrophils, actively participate in tissue injury, repair and remodeling^43,44^. In our study, the analysis of cell composition at high expression positions of related genes revealed that the cell types at these positions were concentrated in fibroblasts, SMCs, mesenchymal–stromal cells, macrophages and endothelial cells. These results are consistent with the identified cellular roles in AAD pathogenesis and support the potential of the three candidate proteins as pathogenic molecules in AAD. Despite advancements in understanding the dynamics of cell populations and ECM remodeling in aortic physiology and pathology, the specific trigger of aortic dissection remains elusive. Future research efforts are needed to determine whether these three molecules are pathogenic and to elucidate their mechanisms of action.

As highlighted in demographic studies, approximately 65% of AAD patients are male, with the condition most commonly occurring in their seventies (mean age 63 years)^10,45,46^. While African American patients with AAD tend to be younger (mean age 54.6 ± 12.8 years) compared to white individuals (mean age 64.2 ± 15.2 years; *P* < 0.001), and are more likely to have histories of cocaine abuse, hypertension and diabetes^47^. This is consistent with our case-control analysis, in which AAD patients presented at a relatively younger age and exhibited a high prevalence of hypertension. Women are less frequently affected by AAD, comprising about 32% of all patients in the IRAD^48^. Observational studies have identified smoking, hyperlipidemia, hypertension and standing height as independent risk factors for AAD development. In observational studies, smoking, hyperlipidemia, hypertension and standing height have been suggested as independent risk factors associated with the development of AAD^42,49^. In our case-control, AAD patients demonstrated higher standing heights and SBP, and a greater prevalence of smoking and hypertension compared to controls. Univariate analysis also indicated that hypertension, smoking, standing height and SBP are associated with an increased risk of AAD, which is consistent with the epidemiologic literature^42,50^.

d-dimer is a degradation product of crosslinked fibrin and a well-established biomarker of coagulation and fibrinolysis^51^. Elevated d-dimer may play an important role in the formation and development of various cardiovascular diseases, especially in pulmonary embolism disease. Multiple studies have shown that exposure of tissue factors in the arterial smooth muscle layer of AAD patients activates exogenous coagulation pathways and increases blood d-dimer levels^52^. Therefore, d-dimer also plays an important role in the diagnosis of aortic dissection. Given the complexity of AAD pathophysiology, single biomarkers are insufficient for reliable diagnosis. We therefore constructed a multi-marker score combining d-dimer, ADAMTS8, age, height, and SBP. In an independent validation set of 393 participants, this score achieved an AUC of 0.928 for distinguishing AAD from healthy controls, which was significantly superior to the single biomarker ADAMTS8 (AUC = 0.788), confirming the stability and reproducibility of the model and ruling out accidental results. More importantly, the three life-threatening acute chest pain conditions (AAD, AMI, and PE) have highly similar clinical symptoms, leading to high misdiagnosis rates in emergency settings. Our multi-marker score exhibited excellent performance in differentiating AAD from other acute chest pain etiologies, with an AUC of 0.887 for distinguishing AAD from all non-AAD chest pain patients. Specifically, it achieved an AUC of 0.913 for differentiating AAD from AMI and 0.825 for distinguishing AAD from PE. Although ADAMTS8 itself had a certain discriminatory ability, the combined score significantly improved diagnostic efficacy, which is of great practical significance for rapid triage in the emergency department and avoiding fatal misdiagnosis. The superiority of the multi-marker score over single indicators lies in the fact that single biomarkers such as ADAMTS8 have limited specificity and sensitivity in complex chest pain etiologies, while the combination of molecular biomarkers and clinical risk factors comprehensively captures the multi-faceted pathophysiological characteristics of AAD, resulting in a substantial improvement in AUC and stable performance in the independent validation set, indicating strong robustness of the model.

A prospective population-based study revealed that only 67.3% of the patients were on antihypertensive medication during the 5 years prior to dissection. Proportion analysis of blood pressure data showed that 61.9% of subsequent blood pressure readings were > 140/90 mmHg, despite most patients receiving combined antihypertensive therapy^53,54^. Various risk factors have been shown to predispose the aorta to become fragile to increase the risk of AAD. Mechanistically, factors such as older age, dyslipidemia, elevated apolipoprotein levels and arterial hypertension can accelerate atherosclerotic degeneration of the aorta. This degeneration manifests as intimal thickening, fibrosis, calcification, extracellular fatty acid deposition and ECM degradation, all of which impair the aortic wall’s elasticity, leading to aortic wall fragility^55,56^. Hypertension also exerts increased pressure on the aortic wall, potentially initiating an intimal tear. It may further stimulate the production of pro-inflammatory cytokines and matrix metalloproteinases, leading to excessive ECM degradation^56^.

Collectively, our findings demonstrate that this multi-marker score, validated in a large independent validation set, enables early and differential diagnosis of AAD and supports timely referral, surgical intervention, or emergency operation. Its ability to distinguish AAD from AMI and PE is particularly valuable in acute care settings. Key strengths of this study include the identification of novel AAD-associated biomarkers (PTMA, CD36, and ADAMTS8), a two-stage design involving model development in a discovery set and rigorous validation in a large independent validation set, excellent performance in differentiating AAD from other life-threatening causes of chest pain, and potential for rapid triage in the emergency department. Further investigation into molecular mechanisms and prospective multicenter studies will help optimize clinical therapeutic strategies and improve patient outcomes.

### Study limitations

This study has several limitations. First, all samples were from two hospitals, but both located in the same region, which may limit the generalizability of our findings. Expanded sample sizes and multi-regional, multi-center study designs are needed to improve statistical power and external validity and to further confirm the diagnostic reliability of our model. Second, although we validated our multi-marker score in a large independent set of 393 subjects, including patients with AAD, AMI, PE, and healthy controls, which strengthened its reliability and clinical applicability, the overall sample size still restricted in-depth subgroup analyses, especially among patients with complex comorbidities. Further multi-center prospective cohorts are therefore required to improve the applicability and robustness of this scoring system. Third, the potential value and biological roles of other candidate biomarkers not included in our diagnostic panel should not be overlooked. In particular, further mechanistic studies, including functional assays and animal models, are needed to clarify the causal roles of PTMA, CD36, and ADAMTS8 in the pathogenesis of AAD. Fourth, protein quantification may vary across different analytical platforms, which could influence the stability of the proposed cutoff values. Targeted proteomic assays may be utilized to improve measurement precision in future investigations. Finally, clinical imaging remains the gold standard for AAD diagnosis, and our scoring model should be used as an adjunctive tool rather than a replacement. Currently, we have designed a multi-center prospective study that is under registration, to further validate the model in diverse populations.

## Conclusions

We identified PTMA, ADAMTS8, and CD36 as novel biomarkers associated with AAD. We developed and validated a five-biomarker risk score that can effectively distinguish AAD patients from healthy controls. More importantly, this multi-marker score shows excellent performance in the differential diagnosis of life-threatening acute chest pain syndromes, including AAD, AMI, and PE. It represents a rapid, sensitive, and cost-effective auxiliary tool for early risk stratification and timely triage in the emergency department. Our findings may provide evidence-based insights for early identification, accurate diagnosis, and optimized clinical management of AAD.

## Supplementary Information

Below is the link to the electronic supplementary material.

Supplementary Material 1. Table S1. List of differentially expressed genes in ST clusters.

Supplementary Material 2. Figure S1. Determination of PCA models and composition of each PCA component. (**A**) Based on the scree plot, the first two principal components were retained. The contribution of the first two principal components to the total variance was 83.9% and 15.7%, respectively, and together they explained approximately 99.6% of the total cumulative variance. (**B**, **C**) Contribution of each variable to the first two principal components (Dim-1, Dim-2): candidate proteins (CD36, ADAMTS8, PTMA) and clinical risk factors or laboratory indicators showed different contribution degrees, with ADAMTS8 and D-dimer having the highest contribution to Dim-1 and Dim-2.

Supplementary Material 3.

## Data Availability

All supporting data are included within the main article and its supplementary files.

## Abbreviations

AAD: Acute aortic dissection
ADAMTS8: A disintegrin and metalloproteinase with thrombospondin motifs 8
AUC: Area under the curve
AMI: Acute myocardial infarction
α-SMA: α-smooth muscle actin
CD31: Cluster of differentiation 31
CD68: Cluster of differentiation 68
CI: Confidence interval
CT: Computed tomography
ECM: Extracellular matrix proteins
GAPDH: Glyceraldehyde-3-phosphate dehydrogenase
HCL: Human cell landscape
IHC: Immunohistochemical
IOD: Integrated optical density
MRI: Magnetic resonance imaging
OR: Odds ratio
PCA: Principal component analysis
PE: Pulmonary embolism patients
PTMA: Prothymosinα
ROC: Receiver operating characteristic
SBP: Systolic blood pressure
SD: Standard deviation
SDS-PAGE: Standard sodium dodecyl sulfate polyacrylamide gel electrophoresis
SMCs: Smooth muscle cells
ST: Spatial transcriptomics
TGF-β1: Transforming growth factor beta1
VSMCs: Vascular smooth muscle cells
