# Supplementary figures and images for: A d-dimer and ADAMTS8 based multi-marker score for the diagnosis of acute aortic dissection
Source: Sci Rep. 2026 May 7;16:14679. doi: 10.1038/s41598-026-51121-w (PMC13156294; doi:10.1038/s41598-026-51121-w)

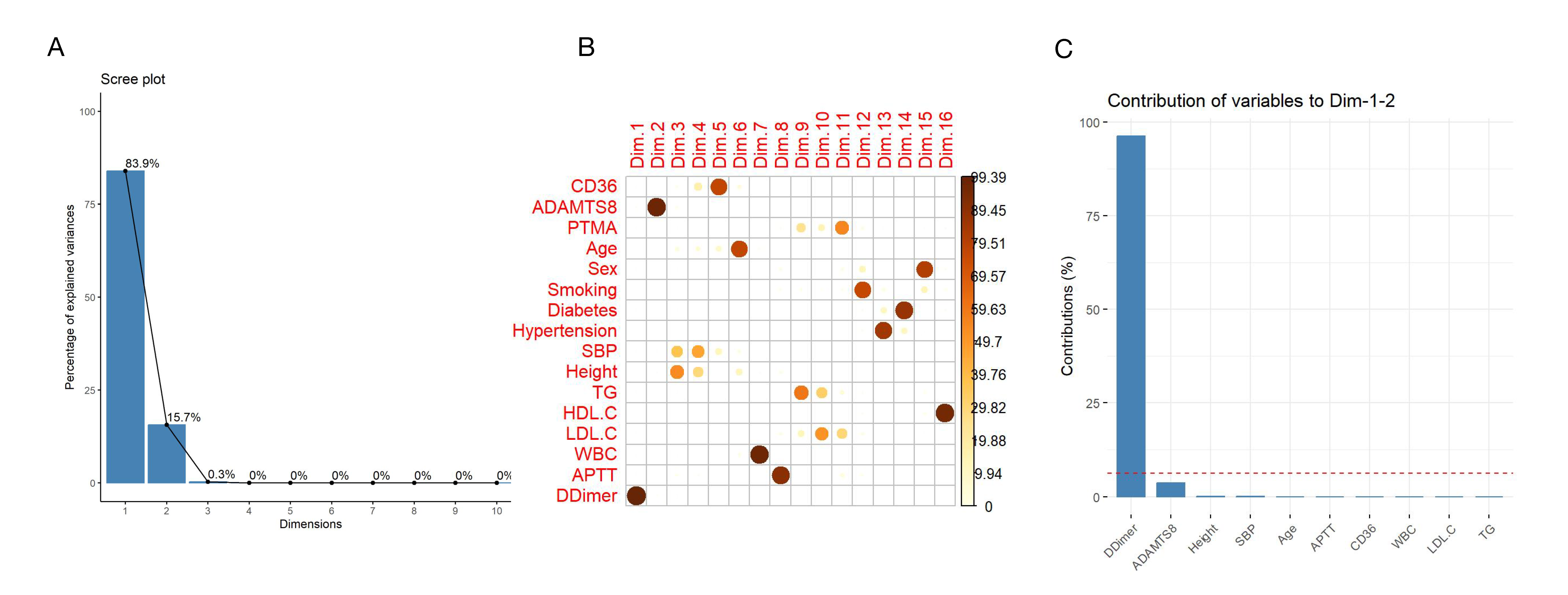

Supplement: Supplementary file 2 — Supplementary Material 2. Figure S1. Determination of PCA models and composition of each PCA component. (A) Based on the scree plot, the first two principal components were retained. The contribution of the first two principal components to the total variance was 83.9% and 15.7%, respectively, and together they explained approximately 99.6% of the total cumulative variance. (B, C) Contribution of each variable to the first two principal components (Dim-1, Dim-2): candidate proteins (CD36, ADAMTS8, PTMA) and clinical risk factors or laboratory indicators showed different contribution degrees, with ADAMTS8 and D-dimer having the highest contribution to Dim-1 and Dim-2. [file 41598_2026_51121_MOESM2_ESM.tiff]
